# Phenotypic detection and genotyping of *Clostridium perfringens* associated with enterotoxemia in sheep in the Qassim Region of Saudi Arabia

**DOI:** 10.14202/vetworld.2021.578-584

**Published:** 2021-03-06

**Authors:** Fehaid Alsaab, Ali Wahdan, Elhassan M. A. Saeed

**Affiliations:** 1 Veterinarian at Ministry of Environment, Water and Agriculture, Kingdom of Saudi Arabia; 2 Department of Bacteriology, Immunology and Mycology, Faculty of Veterinary Medicine, Suez Canal University, Egypt; 3 Department of Veterinary Medicine, College of Agriculture and Veterinary Medicine, Qassim University, Buraydah, Saudi Arabia; 4 Department of Microbiology, Faculty of Veterinary Medicine, Khartoum University, Sudan

**Keywords:** *Clostridium perfringens* toxinotypes, enterotoxaemia, Qassim Region, real-time polymerase chain reaction, sheep, VITEK 2

## Abstract

**Background and Aim:**

Enterotoxemia caused by *Clostridium perfringens* toxinotypes is an often fatal disease of sheep of all ages, with a substantial economic loss to the sheep industry. This study was conducted to isolate *C. perfringens* from suspected cases of enterotoxemia in sheep in the central part of the Qassim Region, Saudi Arabia, and to determine the prevalent toxinotype by detecting alpha (*cpA*), beta (*cpB*), and epsilon (*etX*) toxin genes, which might help control this disease locally.

**Materials and Methods:**

A total of 93 rectal swabs and intestinal content samples were collected from diseased and animals suspected of having died of enterotoxemia in early 2020. Samples were subjected to bacteriological examination, biochemical analysis of isolates by VITEK 2, and molecular toxinotyping of isolates by LightCycler^®^ real-time polymerase chain reaction (RT-PCR).

**Results:**

Our results revealed that only 14 isolates were confirmed by VITEK 2 as being *C. perfringens*, with excellent identification (probability of 95% and 97%). According to the toxinotyping of isolates by RT-PCR, all 14 isolates possessed both the *cpA* and *etX* toxin genes, while the *cpB* toxin gene was not detected in any of the isolates.

**Conclusion:**

Our findings demonstrated that *C. perfringens* type D was the only toxinotype found in the central part of the Qassim Region in 2020; moreover, according to the culture method, only 15% (14/93) of the suspected cases of enterotoxemia were confirmed to be caused by *C. perfringens* infection, which highlighted the importance of clinical and laboratory differential diagnosis of enterotoxemia in sheep.

## Introduction

*Clostridium perfringens*, the causative agent of enterotoxemia, is considered an aero-tolerant anaerobe and a spore-forming, non-motile, and Gram-positive rod. It is a natural intestinal inhabitant in most animal species and in humans; it is found in small numbers and expels a little amount of toxin that is easily removed by the regular intestinal movement [1,2]. However, when the intestinal environment is altered by sudden changes in diet or other factors, *C. perfringens* multiplies and produces powerful toxins that act locally or are absorbed into the circulation, thus constituting the pivotal step in the onset of enterotoxemia and having devastating effects on the host, including colonic convulsions, pasty diarrhea, and nervous signs [1,3]. Depending on its four major toxins, namely, alpha (*cpA*), beta (*cpB*), epsilon (*etX*), and iota (*itX*), *C. perfringens* was previously classified into five toxinotypes, designated A through E, which were recently updated to seven toxinotypes (A–G) [1]. *cpA* is produced by all types, *cpB* is produced by Types B and C, *etX* is produced by Types B and D, while *itX* is produced by Type E [4]. In addition to these four major toxins, several other minor toxins are also ­produced, constituting a collection of more than 20 virulent toxins secreted by this bacterium [1,5]. These toxins vary in pathogenicity and host specificity [6]. Type D, which is known as “overeating disease” or “pulpy kidney,” is the most important and common form of enterotoxemia in sheep [7,8]. *cpA*, *cpB*, *etX*, and enterotoxin were the only toxins reported previously in Saudi Arabia [9]. All ages of sheep are susceptible to these toxins, especially lambs nursed by heavy lactating ewes and lambs weaned on lush pastures or in feedlots [10]. Enterotoxemia has a high fatality rate and, thus, causes considerable economic loss to the sheep industry [11]. The presumptive diagnosis of *C. perfringens* intestinal infections can be established based on history, clinical signs, and postmortem lesions [12]. A definitive diagnosis of this condition can be established through the detection of its toxins by neutralization test in mice or by ELISA in intestinal content and quantitative culture followed by ­genotyping [13]. Polymerase chain reaction (PCR) genotyping provides a useful alternative to *in vivo* toxin neutralization tests for typing *C. perfringens* isolates. Genotypes can, in many cases, provide the final piece of information needed to establish a diagnosis [12]. The control of major sheep diseases in this area of study depends mainly on vaccination. Nine bacterial and viral vaccines, including the enterotoxemia vaccine, are made available by the public sector and are usually delivered on request. The enterotoxemia vaccine is an octavalent inactivated toxoid and anaculture of *C. perfringens* types A−D plus four other clostridial species.

Despite the large number and importance of sheep as a main source of meat, and continuous reports of the occurrence of suspected ovine enterotoxemia at veterinary clinics in the central part of the Qassim Region of Saudi Arabia, data about this disease are scarce.

Therefore, this study was conducted to determine the prevalence and toxinotypes of *C. perfringens* in clinical samples of suspected cases of enterotoxemia in sheep.

## Materials and Methods

### Ethical approval

Live animals were not used in this study; thus, ethical approval was not needed.

### Study period and location

This study was conducted from January to September 2020. Samples were collected from the central part of the Qassim Region of Saudi Arabia. Samples were processed at Microbiology Laboratory of the Veterinary Medicine Department, Qassim University.

### Samples

A total of 93 samples were collected from sheep of different ages that were suspected to have enterotoxemia (50 rectal swabs and 43 intestinal content samples from animals that had died within 6 h) in the central part of the Qassim Region, Saudi Arabia, in the first half of 2020. Samples were obtained from cases attended at the Veterinary Teaching Hospital, College of Agriculture and Veterinary Medicine, Qassim University, other veterinary clinics, and from sheep flocks in the area. Samples were collected in sealed sterile labeled containers and transported to the Microbiology Laboratory of the Veterinary Medicine Department, Qassim University, for investigation.

### Isolation of *C. perfringens*

Samples were first enriched in cooked meat medium (CMM) (Difco Laboratories, Becton Dickinson, Sparks, MD, USA) and then streaked on 5% Blood Agar (BA) (Oxoid, Ltd, Basingstoke, UK), as per standard procedures. Both inoculated media were incubated anaerobically at 40°C for 48 h using a 3.5 L anaerobic jar (Oxoid Ltd) equipped with a GasPak™ Anaerobe Container System for anaerobiosis (Becton, Dickinson & Co., Sparks, USA), according to the manufacturer’s instructions and as described previously [14]. After the incubation period, colonies with cultural properties consistent with those of *C. perfringens* [15] were subcultured onto BA until purity was achieved. In addition to growth characteristics, suspected *C. perfringens* isolates were identified based on their cellular morphology in Gram-stained smears [15]. Purified colonies were grown anaerobically and kept in CMM for the biochemical confirmation and genotyping of isolates.

### Biochemical confirmation of isolates

The obtained isolates were confirmed biochemically using a VITEK 2 ANC system (bioMérieux, Marcy l’Etoile, France) [16]. A suspension from each isolate was prepared after overnight anaerobic growth on BA plates. An inoculating loop was used to transfer sufficient fresh colonies onto a sterile plastic tube containing 3 mL of saline. The suspension was adjusted to a McFarland standard of 2.70-3.30 using a Densicheck (bioMérieux). The tube suspensions and ANC cards were placed in a VITEK 2 cassette and introduced into the VITEK 2 machine for testing. Eventually, the identities of the isolates were obtained with the aid of the accompanying software program.

### Toxin genotyping of *C. perfringens* isolates

DNA was extracted from the biochemically confirmed *C. perfringens* isolates using a PureLink^®^ Genomic DNA Kit (Invitrogen, Life Technologies, USA), according to the manufacturer’s instructions.

Uniplex real-time PCR was performed for the detection of single toxin genes using the primers and probes illustrated in Table-1 [16], which are specific for the *cpA*, *cpB*, and *etX* *C. perfringens* toxin genes. The primers and probes were obtained from Metabion (Germany). PCR was conducted on a LightCycler^®^ Carousel-Based System apparatus (Roche Applied Science, Mannheim, Germany) using the cycling conditions provided in Table-2, as described ­previously [17]. The PCR reaction mixture (total volume, 20 mL) contained the forward and reverse primers, probe, light cycler FastStart DNA Master Plus 5× buffer (Roche Diagnostics GmbH, Germany; prepared by pipetting 10 mL from the enzyme vial into the 5× master mix vial after vortexing, followed by the centrifugation of the two vials), template DNA, and PCR grade water (Table-3). For negative and positive controls, template DNA was replaced by water and the DNA of *C. perfringens* ATCC 19574 reference strain was used, respectively.

**Table-1:** Sequences of primers and probes used for typing of *Clostridium perfringens cpA*, *cpB,* and *etX* toxin genes [16].

Toxin gene	Sequences
*cpA*	F-TGCACTATTTTGGAGATATAGATAC
	R-CTGCTGTGTTTATTTTATACTGTTC
	Pr-FAM-TCCTGCTAATGTTACTGCCGTTGA-TAMRA
*cpB*	F-ATTTCATTAGTTATAGTTAGTTCAC
	R-TTATAGTAGTAGTTTTGCCTATATC
	Pr-HEX-AACGGATGCCTATTATCACCAACT-TAMRA
*etX*	F-TTAACTAATGATACTCAACAAGAAC
	R-GTTTCATTAAAAGGAACAGTAAAC
	Pr-FAM-TGCTTGTATCGAAGTTCCCACAGT-TAMRA

*cpA*=Alpha, *cpB*=Beta,* etX*=Epsilon

**Table-2:** Cycling conditions of LightCycler^®^ real-time polymerase chain reaction for the detection of *Clostridium perfringens* toxin genes.

Target gene	Initial denaturation	Quantification amplification (40-45 cycles)		Cooling

		Denaturation	Annealing and extension	
*cpA*	95°C 10 min	95°C 30 s	55°C 1 min	40°C 30 s
*cpB*	95°C 10 min	95°C 30 s	55°C 1 min	40°C 30 s
*etX*	95°C 10 min	95°C 30 s	55°C 1 min	40°C 30 s

*cpA*=Alpha, *cpB*=Beta,* etX*=Epsilon

**Table-3:** Components and quantities of Uniplex real-time PCR total reaction volume.

Component	Volume (μL)
Master Mix (5×)	4
Water, PCR grade	8
Primer, F. (20 pmol)	1
Primer, R. (20 pmol)	1
Probe	1
DNA*	5
Total	20

* For negative and positive controls, template DNA was replaced by PCR water and DNA of *Clostridium perfringens* ATCC 19574 reference strain, respectively. PCR=Polymerase chain reaction

## Results

### Isolation rate of *C. perfringens*-like colonies

A total of 40 *C. perfringens*-like colonies were recovered from the 93 collected samples, with a prevalence rate of 43%. Regarding the rectal swabs, the isolation rate of *C. perfringens*-like colonies was 32% (16/50), while that of intestinal content was 55.8% (24/43).

### Morphological identification of *C. perfringens* isolates

**Figure-1:**
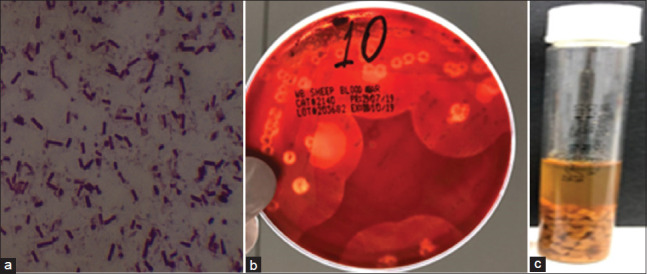
(a) Microscopical appearance of Gram-stained *Clostridium perfringens* bacilli; (b) colonies of *C. perfringens* demonstrating double zone of hemolysis on sheep blood agar; (c) turbid growth and saccharolytic activity of *C. perfringens* in cooked meat medium.

The suspected colonies were identified based on characteristic colony morphology and Gram staining. The Gram-stained smears from the purified colonies revealed the presence of Gram-positive rods arranged singly or in pairs, thick, and straight-sided (Figure-1a). The recovered colonies were round, smooth, glistening, and exhibited a double zone of hemolysis on sheep BA (Figure-1b). In CMM, cultures of *C. perfringens* showed turbidity and red color, indicating saccharolytic activity (Figure-1c).

### Biochemical characterization of the isolates

All 40 recovered isolates were examined biochemically using a VITEK 2 compact system. The results obtained revealed that only 14 isolates were confirmed as being *C. perfringens* by the ANC card, with excellent identification (probability, 95% and 97%). This finding indicated that only 15% (14/93) of our samples were confirmed as being positive for *C. perfringens*. The confirmed isolates were distributed across sample types and areas of study (Table-4). The confirmed isolation rate from samples of intestinal contents was much higher than that obtained for rectal swabs (25.6%, 11/43; and 6%, 3/50, respectively). However, the difference in the isolation rates according to locations was negligible (22.2%, 2/9; 17.4%, 4/23; and 13.1%, 8/61 for the Veterinary Teaching Hospital, sheep flocks, and other veterinary clinics in the area of study, respectively).

**Table-4:** Prevalence of *Clostridium perfringens* and its toxin genes in different locations and types of samples from sheep with suspected enterotoxaemia.

Location	Number of isolates positive by VITEK 2		*Clostridium perfringens* toxin genes detected by real-time polymerase chain reaction					

			Alpha		Beta		Epsilon	

			Sample type							

	Intestinal contents	Rectal swabs	Intestinal contents	Rectal swabs	Intestinal contents	Rectal swabs	Intestinal contents	Rectal swabs
Veterinary Teaching Hospital (n = 9)	2	0	2	0	0	0	2	0
Veterinary clinics (n = 61)	6	2	6	2	0	0	6	2
Sheep flocks (n = 23)	3	1	3	1	0	0	3	1
Total (n = 93)	11	3	11	3	0	0	11	3
	14		14		0		14	

### Toxin genotyping of the confirmed isolates

**Figure-2:**
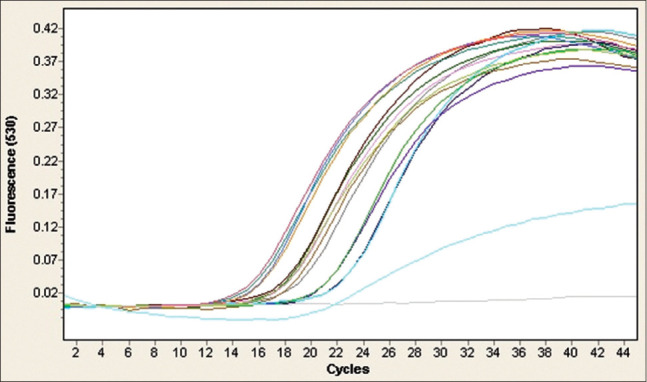
Real-time polymerase chain reaction (PCR) amplification plots for the detection of alpha (*cpA*) toxin gene in 14 isolates of *Clostridium perfringens* obtained from rectal and intestinal content samples from cases of enterotoxaemia in sheep. Negative and positive controls are represented by PCR water and DNA of *C. perfringens* ATCC 19574 reference strain, respectively. All of the 14 *C. perfringens* isolates were positive for *cpA* gene.

**Figure-3:**
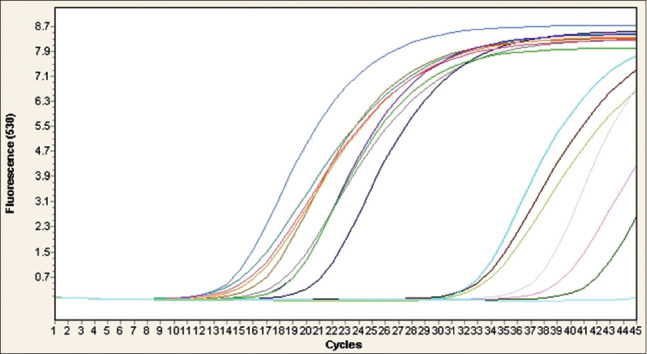
Real-time polymerase chain reaction (PCR) amplification plots for the detection of epsilon (*etX*) toxin gene in 14 isolates of *Clostridium perfringens* obtained from rectal and intestinal content samples from cases of enterotoxaemia in sheep. Negative and positive controls are represented by PCR water and DNA of *C. perfringens* ATCC 19574 reference strain, respectively. All of the 14 *C. perfringens* isolates were positive for *etX* gene.

**Figure-4:**
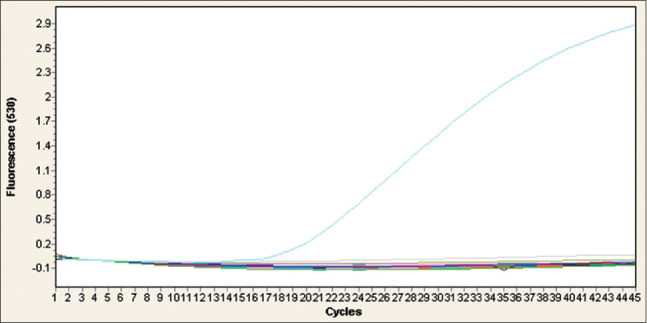
Real-time polymerase chain reaction (PCR) amplification plots for the detection of beta (*cpB*) toxin gene in 14 isolates of *Clostridium perfringens* obtained from rectal and intestinal content samples from cases of enterotoxaemia in sheep. Negative and positive controls are represented by PCR water and DNA of *C. perfringens* ATCC 19574 reference strain, respectively. All of the 14 *C. perfringens* isolates were negative for *cpB* gene.

The 14 biochemically confirmed *C. perfringens* isolates were screened by real-time PCR (RT-PCR) for the detection of *C. perfringens*, *cpA*, *cpB*, and *etX* toxin genes. The results obtained revealed that the *cpA* and *etX* toxin genes were detected in all of the isolates, while the *cpB* toxin gene was not detected in any of the isolates (Figures-2-4 and Table-4). This outcome indicated that *C. perfringens* Type D was the only toxinotype present in the area at the time of the study.

## Discussion

Sheep are the most important animal species in Saudi Arabia, as they are the main source of meat production. However, they suffer from serious diseases, most notably enterotoxemia caused by *C. perfringens*, which is considered one of the highest risk factors in this industry [11]. Thus, the present study was undertaken to determine the association rate of *C. perfringens* and its toxinotypes with suspected enterotoxemia in sheep in the central part of the Qassim Region to aid in the development of a prevention strategy for the disease locally. There are only three previous studies from neighboring and remote regions of the Kingdom of Saudi Arabia that addressed this problem [9,18,19]. One of them [9] included samples from the Qassim Region collected during 2014-2015; however, this region is vast, with an area of 58,046 km²; therefore, additional studies are needed.

The findings of this study revealed that Type D is the main *C. perfringens* toxinotype in the central part of the Qassim Region. This result was obtained based on typical bacterial cell morphology using the Gram staining method, growth and colony properties, confirmation of isolates using a VITEK 2 system, and toxin genotyping of isolates by RT-PCR. This outcome was not unexpected because *C. perfringens* Type D is the main cause of enterotoxemia in sheep, which is known as pulpy kidney or overeating disease [20]. It has been previously found as the most prevalent toxinotype (45.76%, 54/118 isolates) [21]. Moreover, many previous reports [9,18,22-25] have found Type D as the second most prevalent type, after Type A. This disease condition occurs after a sudden change in the type of diet or an increase in the amount of food intake [3]. Such bad management practices are common among sheep flocks in the area of the current study, a fact that justifies the dominant existence of *C. perfringens* Type D. However, most previous studies have reported *C. perfringens* Type A as being the most ubiquitous and prevailing toxinotype [9,18,22-25]. Other types (B, C, and E) are previously reported with varying prevalence. The failure to isolate types other than Type D could be attributed to their existence in small amounts, below the sensitivity level of the culture method, especially for type A, or to their absence (B, C, and E). Moreover, the presence of faster-growing or swarming bacteria in some of our samples may have played a role in this outcome. This is in accord with several previous studies that failed to isolate Types B, C, and E from sheep and goats in Kashmir, India [25], as well as lambs and kids in Italy [11]. However, the use of more sensitive methods, such as direct PCR or toxin detection by ELISA, may lead to the detection of these toxinotypes [9,19,24].

A total of 40 suspected *C. perfringens* isolates were recovered in this study from the 93 collected samples; however, only 14 of them were confirmed by the VITEK 2 system as being *C. perfringens*. This unexpected result may be attributed to impure colonies or contamination, especially by facultative or strict anaerobic swarming bacteria, such as *C. tetani*, which can form a very fine film of swarming growth that is undetectable by the naked eye [26], or to the presence of other organisms that share morphological and growth properties that are similar to those of *C. perfringens*. This low confirmed isolation rate (15%, 14/93) could also be attributed to non-specific clinical signs on which these suspected enterotoxemia cases of sheep are included. At some veterinary clinics, it is a common practice to suspect enterotoxemia based on diarrhea alone. However, diarrhea may be caused by other bacteria, viruses, protozoa, or even non-microbial causes [27]. It might also be noted that our samples were collected during a period of locally limited outbreaks. The current low isolation rate of *C. perfringens* indicates the importance of clinical and laboratory differential diagnosis. A similar isolation rate (14%, 34/240) has been previously recorded in the Eastern Region of Saudi Arabia [19]. Moreover, a recent report [28] from Saudi Arabia found that the prevalence of enterotoxemia was lowest in sheep (21.4%) compared with cattle, goats, and camels. Furthermore, a rate of 10.2% (87/849) has been reported in Iran [22]. In contrast, several previous studies reported higher isolation rates [21,23,25].

In the current study, rectal fecal samples from diarrheal cases (n=50) and intestinal content samples (n=43) were used for the isolation and genotyping of *C. perfringens*. A higher isolation rate was obtained from intestinal content samples (78.6%, 11/14) compared with fecal samples (21.4%, 3/14). This result is in high agreement with previous works [25,28,29].

Genotyping was performed using specific primers and probes for the *cpA*, *cpB*, and *etX* toxin genes of *C. perfringens* [17] (Table-1). The biochemically confirmed isolates (n=14) were screened for the presence of these three genes by RT-PCR. All isolates were positive for both the *cpA* and *etX* toxin genes and none of them was positive for the *cpB* toxin gene (Figures-2-4). This finding indicated that all of isolates were *C. perfringens* Type D. The other toxinotypes of *C. perfringens* (A, B, C, and E) were not detected, based on the fact that Type A produces *cpA* but not *etX*, Types B and C produce *cpB*, which was not detected, and Type E does not produce *etX*, which was detected [1]. Although toxinotypes other than Type D were not detected in the current study, their presence cannot be ruled out because this study was preliminary and based on small sample size. Moreover, Types A, B, and C, but not E, were present in several neighboring and remote regions of Saudi Arabia [18,19,28], with much lower prevalence rates reported for Types B and C compared with Types A and D.

## Conclusion

This research work revealed that *C. perfringens* toxinotype D was the main cause of enterotoxemia in the area of study; therefore, it should be considered in the selection of vaccines to protect against this disease. Furthermore, the low rate of isolation of *C. perfringens* (15%) from suspected enterotoxemia cases stresses the importance of clinical and laboratory differential diagnosis for this disease.

## Authors’ Contributions

FA carried out the study. AW provided technical support and assisted in drafting the manuscript. EMAS planned, designed and supervised the study, and critically revised the final draft of the manuscript. All authors have read and approved the final manuscript.
